# The protective effect of baicalin against renal ischemia-reperfusion injury through inhibition of inflammation and apoptosis

**DOI:** 10.1186/1472-6882-14-19

**Published:** 2014-01-13

**Authors:** Miao Lin, Long Li, Liping Li, Gaurab Pokhrel, Guisheng Qi, Ruiming Rong, Tongyu Zhu

**Affiliations:** 1 Department of Urology, Fudan University Zhongshan Hospital, Shanghai, China; 2 Shanghai Key Laboratory of Organ Transplantation, Shanghai, China

**Keywords:** Baicalin, Ischemia-reperfusion, Kidney, Inflammation, Apoptosis

## Abstract

**Background:**

Renal ischemia-reperfusion injury (IRI) increases the rates of acute kidney failure, delayed graft function, and early mortality after kidney transplantation. The pathophysiology involved includes oxidative stress, mitochondrial dysfunction, and immune-mediated injury. The anti-oxidation, anti-apoptosis, and anti-inflammation properties of baicalin, a flavonoid glycoside isolated from *Scutellaria baicalensis*, have been verified. This study therefore assessed the effects of baicalin against renal IRI in rats.

**Methods:**

Baicalin was intraperitoneally injected 30 min before renal ischemia. Serum and kidneys were harvested 24 h after reperfusion. Renal function and histological changes were assessed. Markers of oxidative stress, the Toll-like receptor (TLR)2 and TLR4 signaling pathway, mitochondrial stress, and cell apoptosis were also evaluated.

**Results:**

Baicalin treatment decreased oxidative stress and histological injury, and improved kidney function, as well as inhibiting proinflammatory responses and tubular apoptosis. Baicalin pretreatment also reduced the expression of TLR2, TLR4, MyD88, p-NF-κB, and p-IκB proteins, as well as decreasing caspase-3 activity and increasing the Bcl-2/Bax ratio.

**Conclusions:**

Baicalin may attenuate renal ischemia-reperfusion injury by inhibiting proinflammatory responses and mitochondria-mediated apoptosis. These effects are associated with the TLR2/4 signaling pathway and mitochondrial stress.

## Background

Renal ischemia-reperfusion injury (IRI), a proinflammatory pathophysiological process, can increase the rates of acute kidney failure, delayed graft function, and early mortality in patients undergoing kidney transplantation [1]. Baicalin is a flavonoid glycoside isolated from *Scutellaria baicalensis*[2]. This root, which has anti-bacterial and anti-inflammatory properties, is a traditional Chinese herb widely used in the treatment of infectious and inflammatory diseases. Baicalin has been shown to possess these protective properties as well, including against various inflammatory diseases [3-5]. Baicalin has also been shown to protect against IRI in various organs, including the heart [6], liver [7], and brain [8], because of its anti-oxidative, anti-inflammatory, and anti-apoptotic effects. However, its impact on renal IRI remains unknown.

Baicalin may protect against IRI by altering the production of various mediators, including reactive oxygen species (ROS), Toll-like receptor (TLR)2 and TLR4, NF-κB, Bax, and Bcl-2. Oxidative stress is significantly ameliorated after baicalin treatment [9,10]. Baicalin has been shown to down-regulate the expression of TLR2, enhance the expression of Bcl-2, and inhibit the activities of Bax and caspase-3 in mastitis [11]. Moreover, baicalin was found to reduce the expression of TLR2/4 and NF-κB, thus inhibiting the TLR2/4 signaling pathway [12].

Oxidative stress, the TLR2/4 signaling pathway, and mitochondrial stress play important roles in renal IRI [13,14]. ROS production during reperfusion is thought to be the main reason for uncontrolled oxidative stress, which leads to changes in expression of Bcl-2 and Bax, which are markers of mitochondrial dysfunction [15]. Renal injuries result in the release of heat shock proteins, high mobility group box 1, and other breakdown products, which subsequently bind to TLRs 2 and 4 as endogenous ligands and induce active immune-mediated injury in damage-associated molecular patterns. TLR activation results in an intracellular cascade of events, during which MyD88-dependent signaling leads to the release of NF-κB from IκB, allowing NF-κB translocation from the cytoplasm to the nucleus, where it mediates the increased expression of inflammatory cytokine genes, leading to a proinflammatory response [16,17]. The direct loss of renal function after IRI is associated with the apoptosis of tubular epithelial cells (TECs), a process that can be induced by increased expression of proinflammatory cytokines or mitochondrial dysfunction.

The close association between the mechanisms of renal IRI and baicalin protection suggested that baicalin may be effective in protecting against renal IRI. To test this hypothesis, we used a rat model of renal IRI. The aims of this study were: (1) to confirm that baicalin treatment protects against renal IRI; (2) to investigate the effects of baicalin on the expression of TLR2 and TLR4 and subsequent inflammatory responses; and (3) to assess the effects of baicalin on mitochondrial dysfunction and the apoptosis of TECs.

## Methods

### Experimental animals

Male Wistar rats weighing 200–250 g were housed in a local facility for laboratory animal care and fed a standard diet and water, according to local ethical guidelines. This study was approved by the Bioethics Committee of Zhongshan Hospital, Fudan University, Shanghai, and adhered to generally accepted international standards.

### Renal ischemia-reperfusion model

Rats were randomly divided into five groups of six rats each: (i) sham group; (ii) IR + saline group; (iii) IR + baicalin (1 mg/kg) group; (iv) IR + baicalin (10 mg/kg) group; and (v) IR + baicalin (100 mg/kg) group. Renal IRI was induced by clamping the left renal artery for 45 min plus a right nephrectomy [18]. Rats were anesthetized through an intraperitoneal injection of pentobarbital sodium (40 mg/kg body weight). After a median abdominal incision, the left renal arteries were clamped for 45 min with serrefine. After clamp removal, adequate restoration of blood flow was checked before abdominal closure. The right kidney was then removed. Sham-operated animals underwent the same surgical procedure without clamping.

Saline-treated animals received intraperitoneal injections of 1 mL 0.9% sterile NaCl 30 min before renal clamping. Baicalin-treated rats received intraperitoneal injections of baicalin (Sigma), diluted in sterile saline to 1, 10, or 100 mg/kg body weight 30 min before renal clamping. After the operation, the rats were kept on a warming blanket for 12 h with food and water available. All animals were sacrificed 24 h after surgery with an overdose of pentobarbital sodium, and their blood and kidneys harvested.

### Plasma biochemical analysis

Whole blood was centrifuged at 1600 g for 25 min at 4°C to obtain serum. An autobiochemistry instrument (Hitachi 7060) was used to measure the levels of serum creatinine (Scr) and blood urea nitrogen (BUN).

### Histology

Renal tissue samples were fixed in 10% formalin for 24 h and embedded in paraffin. The sections were stained with hematoxylin and eosin and semi-quantitatively graded at 200× magnification for tubular dilation and interstitial expansion with edema, inflammatory infiltrate (TID) on a scale of 0–3, with 0 indicating normal tubulointerstitium, and 1, 2, and 3 indicating mild (≤25%), moderate (>25 to 50%), and severe (>50%) TID, respectively. Twelve randomly selected fields of each sample were each examined by two blinded examiners, with the mean for each kidney recorded [19].

### Detection of apoptosis

Terminal deoxynucleotidyl transferase-mediated dUTP-biotin nick end labeling (TUNEL) assay kit (KeyGEN) was used to detect apoptotic cells according to the manufacturer’s instructions. Positive-control sections were from a hepatocarcinoma. Apoptotic cells were examined at 400× magnification over 20 fields of tubular areas [20].

### Measurement of malondialdehyde content and superoxide dismutase activity

Oxidative stress in the kidneys was assessed by measuring the malondialdehyde (MDA) content and superoxide dismutase (SOD) activity. MDA is a terminal product of lipid peroxidation; its intracellular concentration was determined with commercial kits (Beyotime), which used the thiobarbituric acid method to form a red product with a maximum absorbance at 535 nm. The results are reported as μmol per milligram extracted protein. The activity of SOD in the kidneys was detected using a Total Superoxide Dismutase Assay Kit (Beyotime) and reported as U/mg protein.

### Caspase-3 activity assays

Relative caspase-3 activity in kidney was detected with a caspase-3 colorimetric assay kit (KeyGEN) according to the manufacturer’s instructions. Optical density (OD) was measured at 405 nm with a microplate reader (Bio-Tek).

### Western blot analysis

Cell lysates were prepared from 20 to 40 μg kidney tissue and cytoplasmic protein was obtained by centrifugation at 4°C. Equal aliquots of proteins were separated by SDS-PAGE and transferred onto PVDF membranes. Primary antibodies were added and the membranes incubated at 37°C for 2 h with gently shaking. The primary antibodies included anti-TLR2 and anti-TLR4, both from Abcam; and anti-MyD88, anti-NF-κB, anti-p-NF-κB, anti-IκB, anti-p-IκB, anti-cleaved-caspase-3, anti-caspase-9, anti-Bax, and anti-Bcl-2, all from Cell Signaling Technology. After thorough washing, the membranes were incubated for 1 h at room temperature with peroxidase-conjugated secondary antibodies (Jackson ImmunoResearch). Immunoreactive bands were visualized using an ECL system (Amersham Pharmacia). To control for lane loading, the same membranes were also incubated with anti-β-actin antibody (Epitomics), depending on the molecular weight of target proteins. The signals were quantified by scanning densitometry using a Bio-Image Analysis System (Bio-Rad), with each band normalized relative to the actin band in the same sample. The results from each experimental group were expressed as relative integrated intensity compared with that of the control measured with the same batch.

### Quantitative real-time polymerase chain reaction

Total RNA was extracted from rat kidneys with TRIzol reagent (Invitrogen, Shanghai, China) according to the manufacturer’s instructions. Total RNA (3–5 μg) was transcribed into cDNA by Superscript II reverse transcriptase (Invitrogen) and random primer oligonucleotides (Invitrogen). Our gene-specific primers for rat IL-1β, IL-6, TNF-α, and GAPDH have been described [21]. Real-time quantitative polymerase chain reaction was performed in a Bio-Rad iCycler iQ system in combination with the Absolute QPCR SYBR Green premix (Takara Bio Inc., Otsu, Shiga, Japan). After a hot start (15 min at 95°C), the amplification protocol consisted of denaturation for 1 s at 95°C, annealing for 5 s at 60°C, and extension for 10 s at 72°C for 45 cycles. Expression levels were normalized relative to those of GAPDH in the same samples using the 2 − ΔΔCt method.

### Statistical analyses

Data are presented as mean ± SEM. Results in two groups were compared using two-tailed independent t-tests, and results among three or more groups were compared by one-way analysis of variance). All statistical analyses were performed using SPSS 13.0 (SPSS Inc.), with P < 0.05 considered statistically significant.

## Results

### Baicalin attenuated renal dysfunction, ameliorated renal histologic damage, and decreased oxidative stress induced by IRI

**Figure 1:**
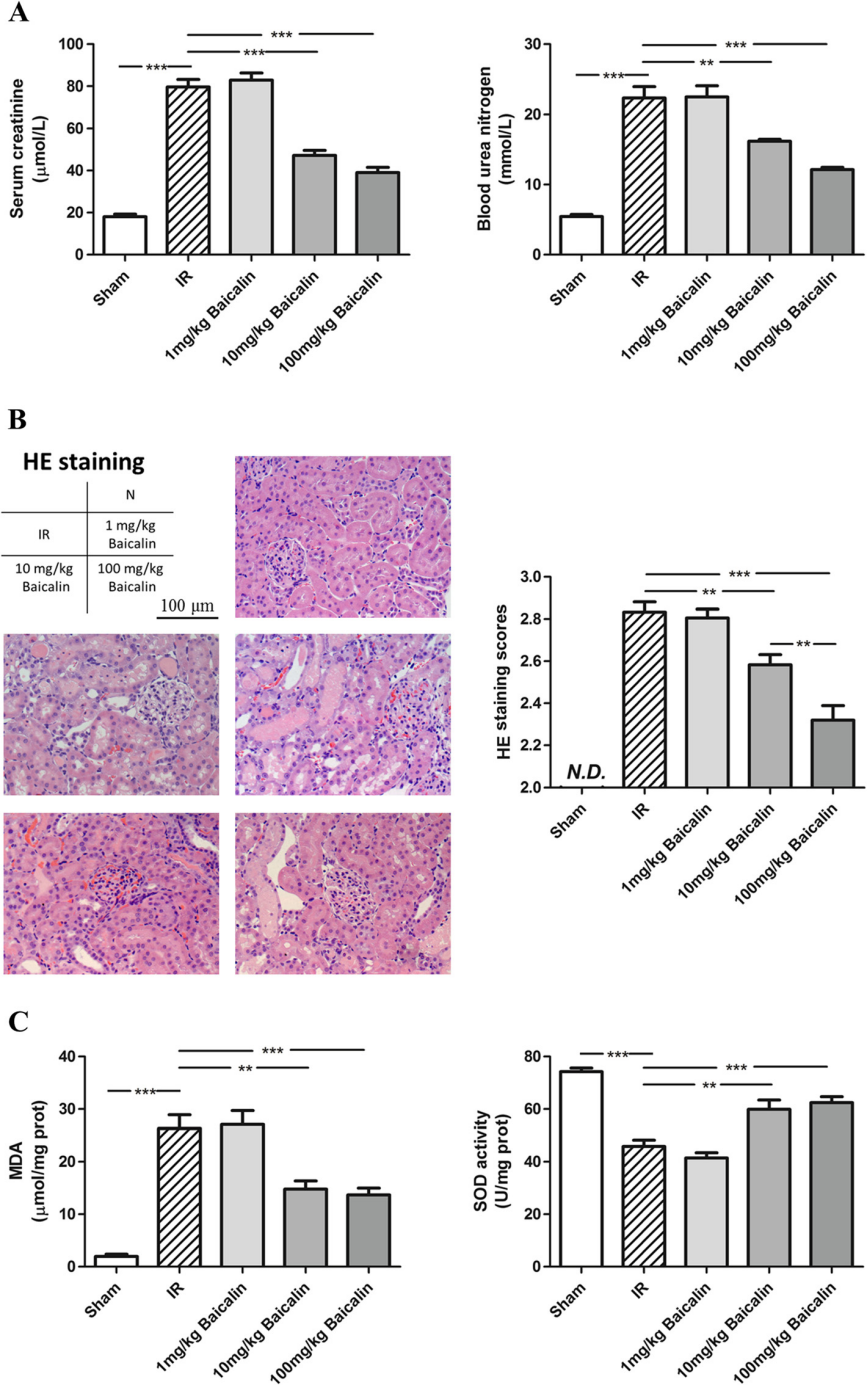
**Effects of baicalin on renal function and histology 24 h after renal IRI. (A)** Serum creatinine and blood urea nitrogen concentrations were significantly higher in the IR + saline than in the sham group. Pretreatment with 10 or 100 mg/kg baicalin dose-dependently inhibited renal dysfunction after renal IRI. **(B)** Baicalin amelioration of histological damage after IRI. **(C)** Baicalin reduction of MDA content and increase of SOD activity, indicating that baicalin reduced oxidative stress. *P < 0.05, **P < 0.01, ***P < 0.001.

Rats that underwent renal IRI had showed significant increases in Scr (79.67 vs. 18.00 μmol/L) and BUN (22.37 vs. 5.45 mmol/L) concentrations compared with the sham operated group. Baicalin pretreatment dose-dependently protected against a loss of renal function, with the two higher doses (10 and 100 mg/kg) significantly decreasing Scr and BUN concentrations (Figure 1A). Histologic examination showed tissue injuries after IRI (Figure 1B), including loss of brush borders, dilation of renal tubules, and urinary cylinders (Additional file 1: Figure S1). Tissue injury, as assessed using a 0–3 point scoring system, was lower for the baicalin treated groups than for the IR + saline group (Figure 1B).

Reoxygenation following ischemia causes tissue oxidative stress, which is considered an important source of IRI. We therefore investigated the effects of baicalin on oxidative stress. Following ischemia reperfusion, MDA content was greatly increased and SOD activity was greatly decreased in kidneys, indicating increases in oxidative stress (Figure 1C). Compared with the sham group, however, MDA content was only slightly up-regulated and the SOD activity was only slightly down-regulated in rats treated with 10 and 100 mg/kg baicalin, indicating that baicalin abrogated the increase in oxidative stress following reperfusion.

### Baicalin down-regulated the TLR2/4, MyD88, and NF-κB signaling, and inhibited subsequent proinflammatory responses

**Figure 2:**
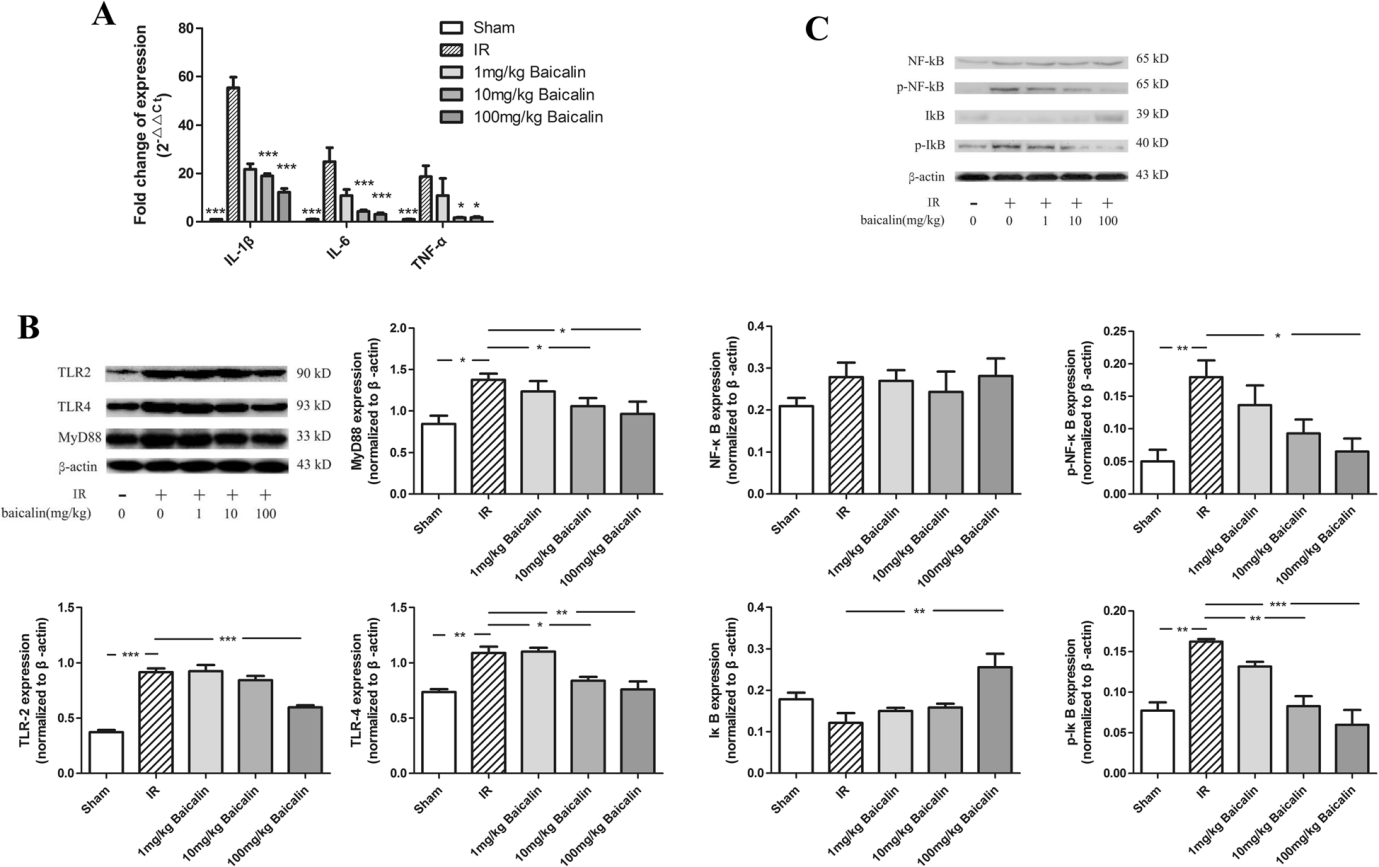
**Effects of baicalin on IRI induced proinflammatory response. (A)** Baicalin inhibition of the IRI induced up-regulation of proinflammatory cytokines. **(B, C)** Activation of the TLR2/4 signaling pathway by IRI, contributing to a proinflammatory response. Pretreatment with baicalin decreased TLR2, TLR4, and MyD88 expression **(B)** and suppressed the phosphorylation of NF-κB and IκB **(C)**, thereby inhibiting related proinflammatory signaling pathways. *P < 0.05, **P < 0.01, ***P < 0.001.

The expression of proinflammatory cytokines (Figure 2A), as well as TLR2/4 expression, were increased in kidneys 24 h after IRI. MyD88, a general adaptor protein of TLR2/4, was also upregulated (Figure 2B). The activation/phosphorylation and nuclear translocation of NF-κB, an important downstream effector of TLR2/4 MyD88-dependent signaling, has been found to enhance proinflammatory responses. Increases in proinflammatory cytokines, including TNF-α and IL-1β, would in turn promote the phosphorylation of NF-κB. We therefore evaluated the levels of phosphorylation of NF-κB and IκB, both of which differed significantly between in the IR + saline and sham groups (Figure 2C). However, the expression of NF-κB was not affected. Baicalin treatment inhibited the increased expression of the proinflammatory cytokines TLR2/4, MyD88, p-NF-κB, and p- IκB, as well as increase the expression of IκB protein, an NF-κB inhibitor, with the degree of inhibition positively related to the dosage of baicalin.

### Baicalin prevented mitochondrial dysfunction, down-regulated the activity of caspase-3, and decreased the apoptosis of TECs following IRI

**Figure 3:**
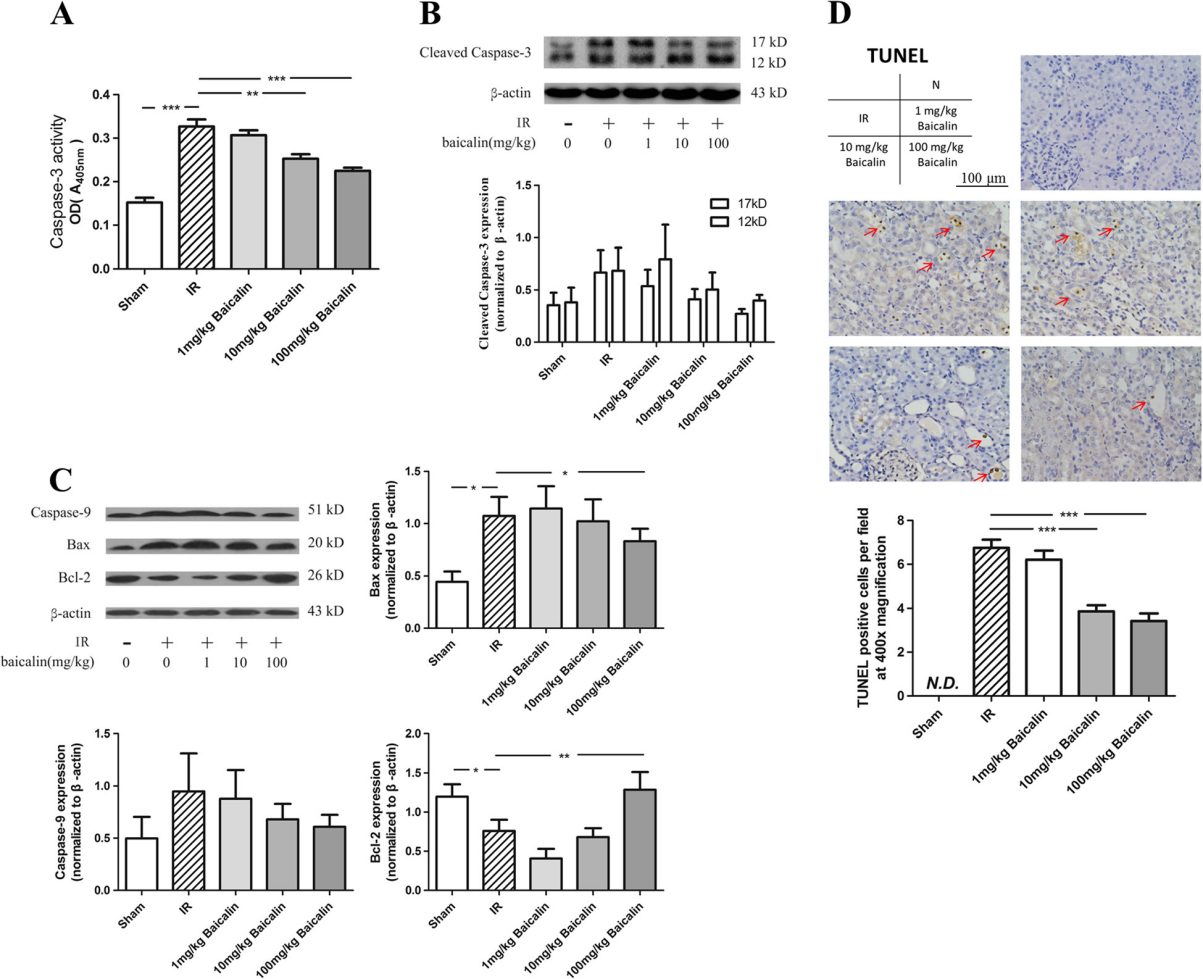
**Effects of baicalin on IRI-induced mitochondrial dysfunction and cell apoptosis. (A, B)** IRI upregulation of the activity and expression of caspase-3 (17 kD, 12 kD) and its inhibition by baicalin. **(C)** Baicalin suppression of the renal IRI-induced increase in mitochondrial stress, as shown by assays of expression of caspase-9, Bax, and Bcl-2. **(D)** TUNEL assay of apoptotic cells. Renal IRI significantly increased the number of apoptotic cells, an increase significantly reduced by treatment with 10 and 100 mg/kg baicalin. *P < 0.05, **P < 0.01, ***P < 0.001.

Baicalin significantly inhibited caspase-3 activity in kidneys 24 h after IRI (Figure 3A), as well as decreasing the expression of cleaved caspase-3 (Figure 3B) 24 h after IRI, compared with the IR + saline group. Western blot assays of caspase-9, Bcl-2, and Bax proteins, which reflect mitochondrial stress, showed that baicalin dose-dependently downregulated the expression of the pro-apoptotic proteins caspase-9 and Bax, while upregulating the expression of the anti-apoptotic protein, Bcl-2 (Figure 3C), indicating that baicalin inhibited mitochondria-mediated apoptosis. TUNEL staining showed that most apoptotic cells were located in tubular areas, with very few in interstitial areas (Figure 3D). Treatment with 10 or 100 mg/kg baicalin significantly reduced the number of apoptotic TECs.

## Discussion

**Figure 4:**
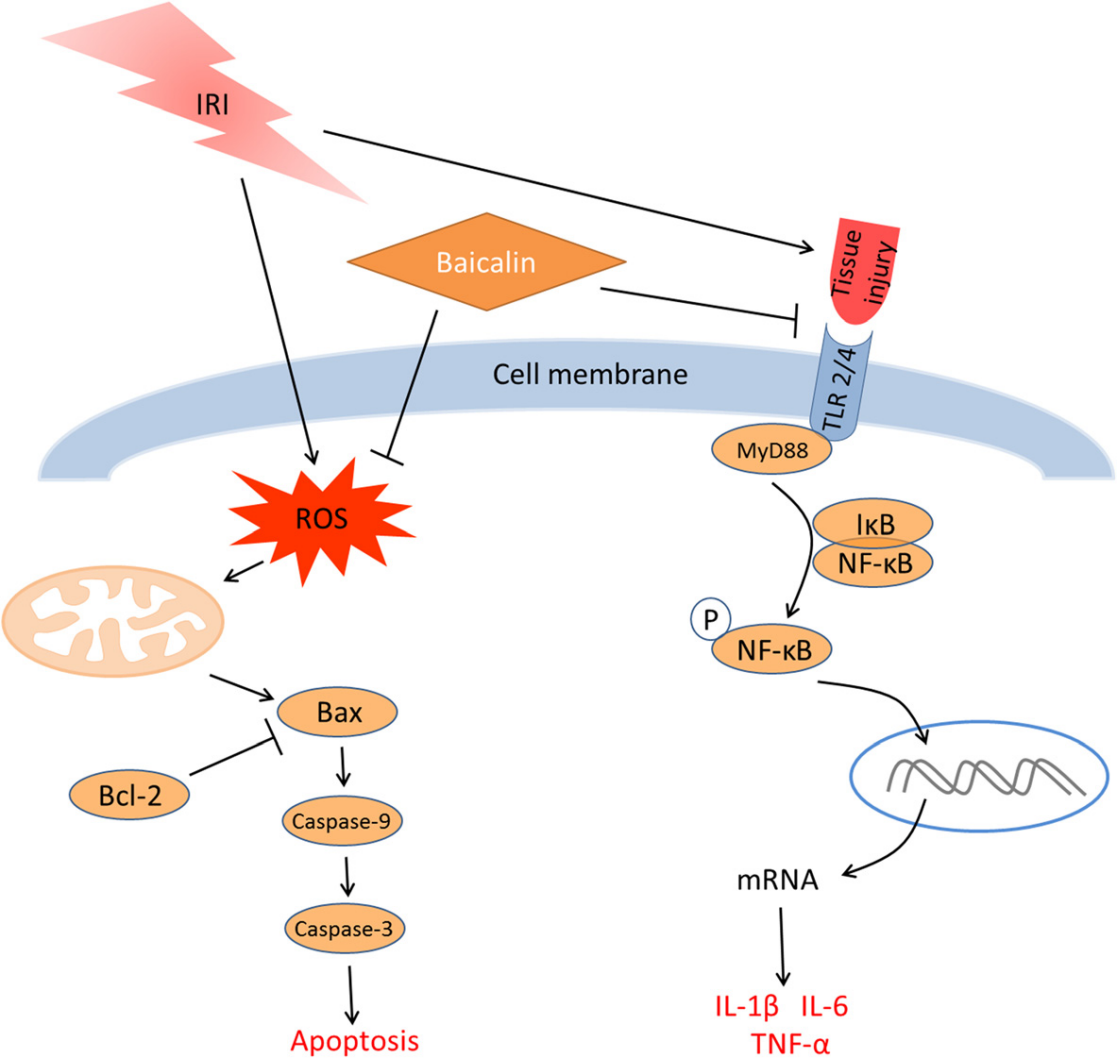
**Schematic diagram of signal pathways involved in renal IRI.** Renal IRI induces oxidative stress and tissue injury, resulting in mitochondrial dysfunction and activation of the TLR2/4 signaling pathway, all of which are targets of baicalin treatment.

To our knowledge, this study is the first to provide in vivo evidence of the potential therapeutic value of baicalin in renal IRI. Baicalin pretreatment decreased oxidative stress and inhibited proinflammatory response and immune-mediated IRI. Moreover, baicalin ameliorated mitochondrial dysfunction and TEC apoptosis. Our findings suggest that the mechanisms underlying this protection are largely due to the inhibition of TLR2/4 and the NF-κB signaling pathway and the deactivation of the mitochondrial stress pathway (Figure 4).

Among the methods tested for attenuation of renal IRI are many drugs [22,23], endocrine hormones [24], erythropoietin [21], and small interfering RNA [25]. Various drawbacks, however, have prevented their clinical application. It’s necessary to find a convenient, effective, and safe method to treat renal IRI. Thus, the efficacy of baicalin against kidney IRI was tested in our experiment.

In evaluating kidney function 24 h after surgery, we found that baicalin pretreatment significantly reduced serum Scr and BUN concentrations compared with the IR + saline group, suggesting that baicalin protects kidneys from IRI. HE staining also showed that baicalin ameliorated pathological damage to the kidneys.

IRI is an antigen-independent inflammatory process that causes tissue damage [26,27]. Kidney IRI has been associated with multiple factors, including endothelial injury, leukocyte infiltration, and tubular epithelial cell activation, all of which trigger and exaggerate inflammation response through the innate and adaptive immune systems [28,29]. The TLR2/4 signaling pathway is an important inflammatory cascade after IRI. TLR2/4 are innate immune receptors on cell surfaces. Myeloid differentiation factor 88 (MyD88), an adaptor protein associated with activation of TLRs, interacts with the IRAK complex, triggering further signaling cascades, including the activation of NF-κB. The nuclear translocation of NF-κB subsequently up-regulates proinflammatory cytokines and chemokines [30]. TLR2 and TLR4 are constitutively expressed in proximal and distal tubules, the thin limb of the loop of Henle and the collecting ducts, with both up-regulated in these sites after IRI [31]. Consistent with these findings, we observed up-regulation of TLR2 and TLR4 proteins in kidney resident cells. The negative regulation of TLR signaling by baicalin has been observed in lipopolysaccharide (LPS)-stimulated human oral keratinocytes [32], oxygen-glucose deprived rat microglial cells [33], and other ischemic organs [34]. Our findings suggest that baicalin treatment decreases TLR2/4 activation and down-regulates the downstream activation of NF-κB signaling in kidneys after IRI.

NF-κB activation can be measured by assays for p-NF-κB and p-IκB. The transcription factor NF-κB, which is inhibited by IκB binding, is involved in the expression of proinflammatory genes [35]. NF-κB in response to IRI is activated via the phosphorylation of IκB, followed by proteasome-mediated degradation. Subsequently, NF-κB undergoes phosphorylation and translocates into the nucleus, where it regulates the proinflammatory responses [36]. Baicalin treatment reduced the expression of p-NF-κB and p-IκB, while increasing the expression of IκB, which inhibits NF-κB. Our results therefore suggest that baicalin inhibited NF-κB activation during IRI.

Proinflammatory cytokines, including IL-1β, IL-6, and TNF-α, are closely associated with renal IRI. High serum concentrations of these cytokines are considered a marker of severity. We found that the expression of these cytokines was significantly increased after IRI, an increase positively related to the activation of TLR2/4 and NF-κB signaling (Figure 4).

Baicalin may also affect oxidative stress after IRI. IRI interrupts the redox balance, which is pivotal for normal kidney function, and results in the accumulation of ROS. Subsequent mitochondrial dysfunction may lead to TEC apoptosis and loss of function [13,15,37]. The mitochondrion is the central organelle in the intrinsic pathway of apoptosis [38,39]. Increased mitochondrial stress during renal IRI up-regulates the Bax/Bcl-2 ratio and activates mitochondria-mediated apoptosis. Increasing the Bax/Bcl-2 ratio will alter mitochondrial membrane permeability, leading to the release of cytochrome C, which, together with caspase-9, forms apoptosomes, subsequently activating a downstream caspase cascade[14]. We found that baicalin down-regulated Bax and up-regulated Bcl-2 expression, further indicating that baicalin inhibits mitochondria-mediated apoptosis (Figure 4). We also found that baicalin downregulated the expression of caspase-9, an important indicator of mitochondria-mediated apoptosis [40].

The results of TUNEL assays showed that apoptosis of TECs was lower in baicalin treated than in IRI + saline-treated animals. Decreased apoptosis is associated with the deactivation of a caspase cascade. Caspase-3 is a downstream effector in this cascade, directly mediating apoptosis when activated by various upstream signals [41], and regarded as a pivotal indicator of apoptosis during IRI [19,42]. We found that baicalin significantly inhibited the activation and activity of caspase-3.

## Conclusions

In summary, our study is the first to show that baicalin pretreatment can significantly ameliorate renal IRI, reduce the expression of proinflammatory cytokines, and inhibit the apoptosis of TECs by inhibiting the TLR2/4 signaling pathway and the mitochondria-mediated cell apoptosis pathway.

## Competing interests

The authors declare that they have no competing interests.

## Authors’ contributions

ML, LL, and LL carried out the molecular biology studies and the immunoassays, analyzed the data, and drafted the manuscript. GP and GQ established the animal models. RR and TZ designed and supervised the study, revised the manuscript, and gave final approval for publication. All authors read and approved the final manuscript.

## Pre-publication history

The pre-publication history for this paper can be accessed here:

http://www.biomedcentral.com/1472-6882/14/19/prepub

## Supplementary Material

Additional file 1: Figure S1 Tissue injuries in renal IRI. Tissue injuries, including loss of brush border, dilation of renal tubules, urinary cylinder were obvious in the IR + saline group. Pretreatment with 10 or 100 mg/kg baicalin reduced tissue injuries. →: bursh border; *: dilation of renal tubules; ▲: urinary cylinder. Click here for file
