# Supplementary figures and images for: The protective effect of baicalin against renal ischemia-reperfusion injury through inhibition of inflammation and apoptosis
Source: BMC Complement Altern Med. 2014 Jan 13;14:19. doi: 10.1186/1472-6882-14-19 (PMC3893527; doi:10.1186/1472-6882-14-19)

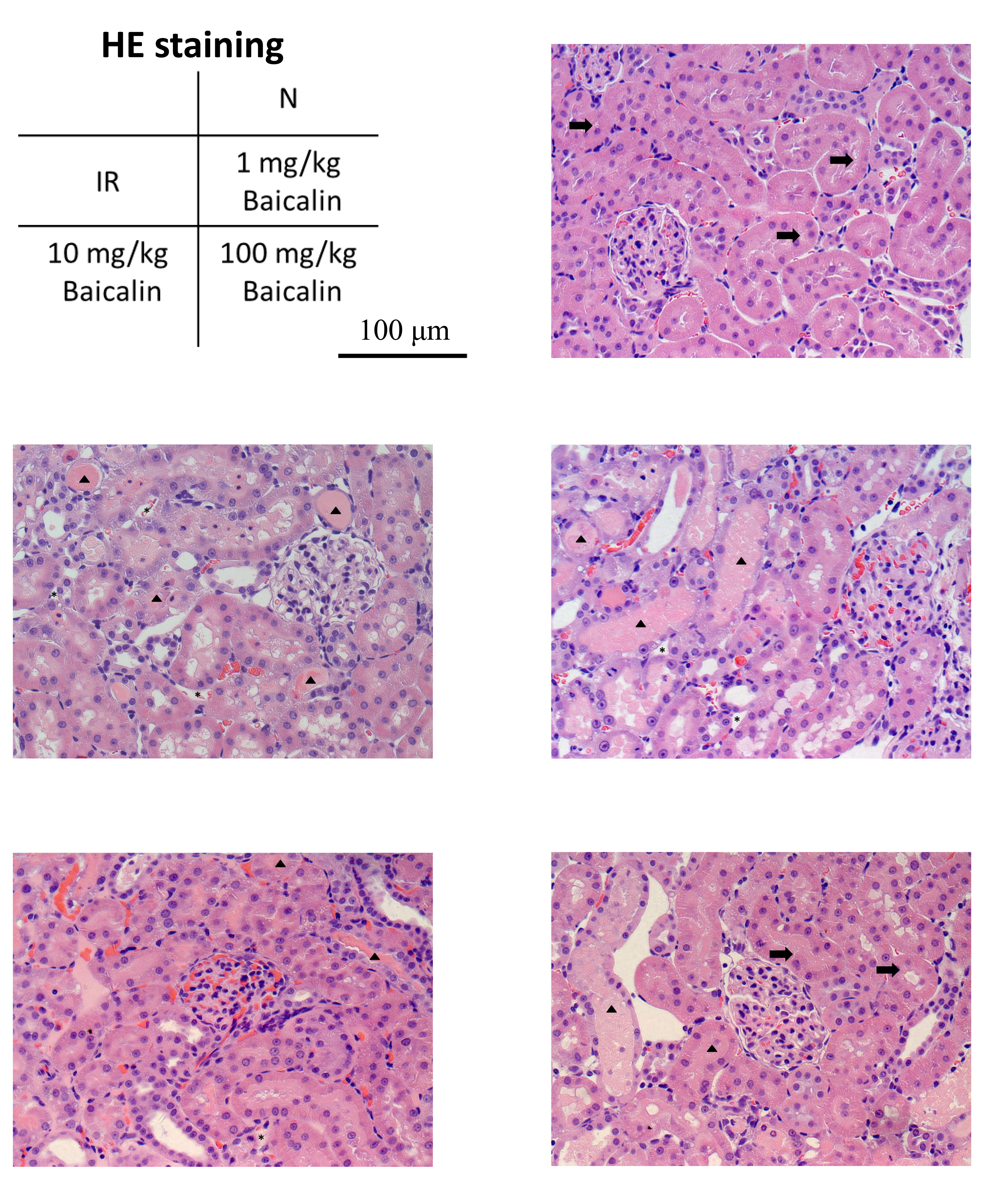

Supplement: Additional file 1: Figure S1 — Tissue injuries in renal IRI. Tissue injuries, including loss of brush border, dilation of renal tubules, urinary cylinder were obvious in the IR + saline group. Pretreatment with 10 or 100 mg/kg baicalin reduced tissue injuries. →: bursh border; *: dilation of renal tubules; ▲: urinary cylinder. [file 1472-6882-14-19-S1.tiff]
